# Glutamine + glutamate level predicts the magnitude of microstructural organization in the gray matter in the healthy elderly

**DOI:** 10.1017/S1041610219001418

**Published:** 2021-01

**Authors:** Tomokazu Motegi, Kosuke Narita, Kazuyuki Fujihara, Masato Kasagi, Yusuke Suzuki, Minami Tagawa, Koichi Ujita, Jamie Near, Masato Fukuda

**Affiliations:** 1 Department of Psychiatry and Neuroscience, Gunma University Graduate School of Medicine, 3-39-22 Showa-machi, Maebashi, Gunma 371-8511, Japan; 2 Department of Psychiatry, Graduate School of Medical Science, Kyoto Prefectural University of Medicine, Kawaramachi-Hirokoji, Kamigyo-ku, Kyoto 602-8566, Japan; 3 Department of Diagnostic Radiology and Nuclear Medicine, Gunma University Graduate School of Medicine, Maebashi, Gunma 371-8511, Japan; 4 Douglas Mental Health University Institute and Department of Psychiatry, McGill University, Montreal, QC, Canada

**Keywords:** diffusion tensor imaging, mean diffusivity, magnetic resonance spectroscopy, glutamine + glutamate (Glx), γ-aminobutyric acid (GABA)

## Abstract

**Background:**

Diffusion tensor imaging (DTI), which is a technique for measuring the degree and direction of movement of water molecules in tissue, has been widely used to noninvasively assess white matter (WM) or gray matter (GM) microstructures *in vivo*. Mean diffusivity (MD), which is the average diffusion across all directions, has been considered as a marker of WM tract degeneration or extracellular space enlargement in GM. Recent lines of evidence suggest that cortical MD can better identify early-stage Alzheimer’s disease than structural morphometric parameters in magnetic resonance imaging. However, knowledge of the relationships between cortical MD and other biological factors in the same cortical region, e.g. metabolites, is still limited.

**Methods:**

Thirty-three healthy elderly individuals [aged 50–77 years (mean, 63.8±7.4 years); 11 males and 22 females] were enrolled. We estimated the associations between cortical MD and neurotransmitter levels. Specifically, we measured levels of γ-aminobutyric acid (GABA) and glutamate + glutamine (Glx), which are inhibitory and excitatory neurotransmitters, respectively, in medial prefrontal cortex (mPFC) and posterior cingulate cortex (PCC) using MEGA-PRESS magnetic resonance spectroscopy, and we measured regional cortical MD using DTI.

**Results:**

Cortical MD was significantly negatively associated with Glx levels in both mPFC and PCC. No significant association was observed between cortical MD and GABA levels in either GM region.

**Conclusion:**

Our findings suggest that degeneration of microstructural organization in GM, as determined on the basis of cortical MD measured by DTI, is accompanied by the decline of Glx metabolism within the same GM region.

## Introduction

Over the last 30 years, various methods of magnetic resonance imaging (MRI) have been developed and used to assess brain alterations associated with normal aging and aged-related neurodegenerative diseases, such as mild cognitive impairment and Alzheimer’s disease (Elman *et al.*, 2017). Diffusion tensor imaging (DTI), a reliable MRI technique for measuring the degree and direction of movement of water molecules in brain tissue, has been widely used to noninvasively assess white matter (WM) or gray matter (GM) microstructures *in vivo* (Le Bihan *et al.*, 1986). Fractional anisotropy (FA), which is thought to be an index of WM integrity calculated by DTI, can reflect the state of neural fibers on the basis of the density, diameter, or coherence of axons (Le Bihan *et al.*, 2001). Mean diffusivity (MD), which is the average magnitude of diffusion across all directions, has been considered as a marker of WM tract degeneration or extracellular space enlargement in GM (Elman *et al.*, 2017; Neil *et al.*, 2002).

Many previous DTI studies have revealed altered FA and MD even in normal aging (Abe *et al.*, 2008; Benedetti *et al.*, 2006; Garcia-Lazaro *et al.*, 2016), age-related neurodegenerative diseases, such as mild cognitive impairment and Alzheimer’s disease (Nesteruk *et al.*, 2016; Nishioka *et al.*, 2015), demyelinating diseases, such as multiple sclerosis (de Kouchkovsky *et al.*, 2016), and neuropsychiatric diseases, such as depression and schizophrenia (Jiang *et al.*, 2017; Singh *et al.*, 2016). Most of these studies have focused on WM or deep GM such as the hippocampus, because these brain regions have high directionality in water diffusion (Manna *et al.*, 2015; Ziyan and Westin, 2008), whereas water diffusion in the cerebral cortex has an isotropic direction at the level of conventional DTI resolution (Elman *et al.*, 2017).

On the other hand, recent lines of DTI evidence have shown that altered cortical MD may reflect the magnitude of microstructural organization in GM, that is, patients with mild cognitive impairment or Alzheimer’s disease have been reported to show increased cortical MD, compared with healthy controls in several GM regions including the posterior cingulate cortex (PCC), entorhinal cortex, amygdala, parahippocampal gyrus, middle temporal gyrus, superior and middle frontal gyri and bilateral supramarginal gyri (Lin *et al.*, 2016; Ray *et al.*, 2006; Rose *et al.*, 2008; Weston *et al.*, 2015). Furthermore, cortical MD begins to increase more generally in middle age (Ni *et al.*, 2010). Noteworthy, a recent twin study showed that cortical MD is associated with genetic factors, distinct from cortical thickness or MD in WM (Elman *et al.*, 2017), suggesting that cortical MD measurement by DTI may have greater potential use for assessing degeneration of microstructural organization in the GM associated with normal aging or age-related diseases than morphometric GM volume estimation in T1-weighted imaging. However, knowledge of relationships between MD and metabolite levels in the same cortical regions is still limited.

In this study of healthy elderly people without dementia, we examined the associations between cortical MD and the levels of γ-aminobutyric acid (GABA) and glutamine + glutamate (Glx), which are inhibitory and excitatory neurotransmitters, respectively, in the medial prefrontal cortex (mPFC) and PCC by DTI and magnetic resonance spectroscopy (MRS) using MEGA-PRESS (Bauer *et al.*, 2013; Jocham *et al.*, 2012). Decreased levels of GABA and Glx in several cortical areas including mPFC and PCC have been reported to be associated with aging (Goryawala *et al.*, 2016; Grachev *et al.*, 2001) and to be found in patients with mild cognitive impairment and Alzheimer’s disease (Antuono *et al.*, 2001; Huang *et al.*, 2016; Riese *et al.*, 2015), that is, mPFC or PCC has been recognized as one of the most important brain areas associated with memory loss or cognitive decline in elderly people. In addition, decreased Glx levels in certain GM regions have been suggested to be associated with neuronal dysfunction and loss in that GM region (Segovia *et al.*, 2001). We hypothesized that cortical MD in mPFC and PCC correlates negatively with the levels of GABA and Glx in the same volume of interest (VOI).

## Methods

### Subjects

Thirty-three individuals [aged 50–77 years (mean, 63.8±7.4 years); 11 males and 22 females] were enrolled in this study (Table 1) on the basis of the following exclusion criteria: history of dementia, neurological or psychiatric illness, diabetes mellitus, chronic alcoholism, smoking, and obesity with a body mass index above 25. All subjects were right-handed, as assessed using the Edinburgh Handedness Inventory (Oldfield, 1971), and provided their written informed consent. The study protocol was approved by the Ethics Committee of Gunma University.

**Table 1.** Demographic characteristics of study subjects

male : female (*n*)	11 : 22
Age (y)	63.8±7.4
Education (y)	14.0±1.9
MOCA (point)	26.3±2.6
JART—predicted full-scale IQ (point)	112.2±7.6
Cambridge Neuropsychological Test Automated Battery	
Spatial recognition memory (percent correct)	81.9±10.8
Rapid Visual Information Processing (A’)	0.9±0.0

MoCA, the Montreal Cognitive Assessment; JART, Japanese version of National Adult Reading Test; A’, signal detection measures of accuracy, Mean ± SD.

### Psychological measurements

The Montreal Cognitive Assessment (MoCA) was administered to all subjects for the screening of cognitive function (Ihara *et al.*, 2013; Nasreddine *et al.*, 2005); although several cut-off points have been proposed for MoCA, we used scores ≥ 20 to exclude subjects with dementia and cognitive impairment, in accordance with a previous report (Waldron-Perrine and Axelrod, 2012). The Japanese version of the National Adult Reading Test was then conducted to estimate premorbid IQs (Matsuoka *et al.*, 2006; Nelson, 1982). The Cambridge neuropsychological test automated battery [CANTAB (Morris *et al.*, 1987); Cambridge Cognition Ltd., Cambridge, United Kingdom] was also administered to each subject of this study and consisted of the following: the Spatial Recognition Memory (SRM) test of visual spatial memory in a two-choice forced discrimination paradigm and the Rapid Visual Information Processing (RVIP) test, which is a visual continuous performance task using digits instead of letters.

### Acquisition of MRS and DTI and T1-weighted anatomical imaging data

#### MRS acquisition

**Figure 1.**
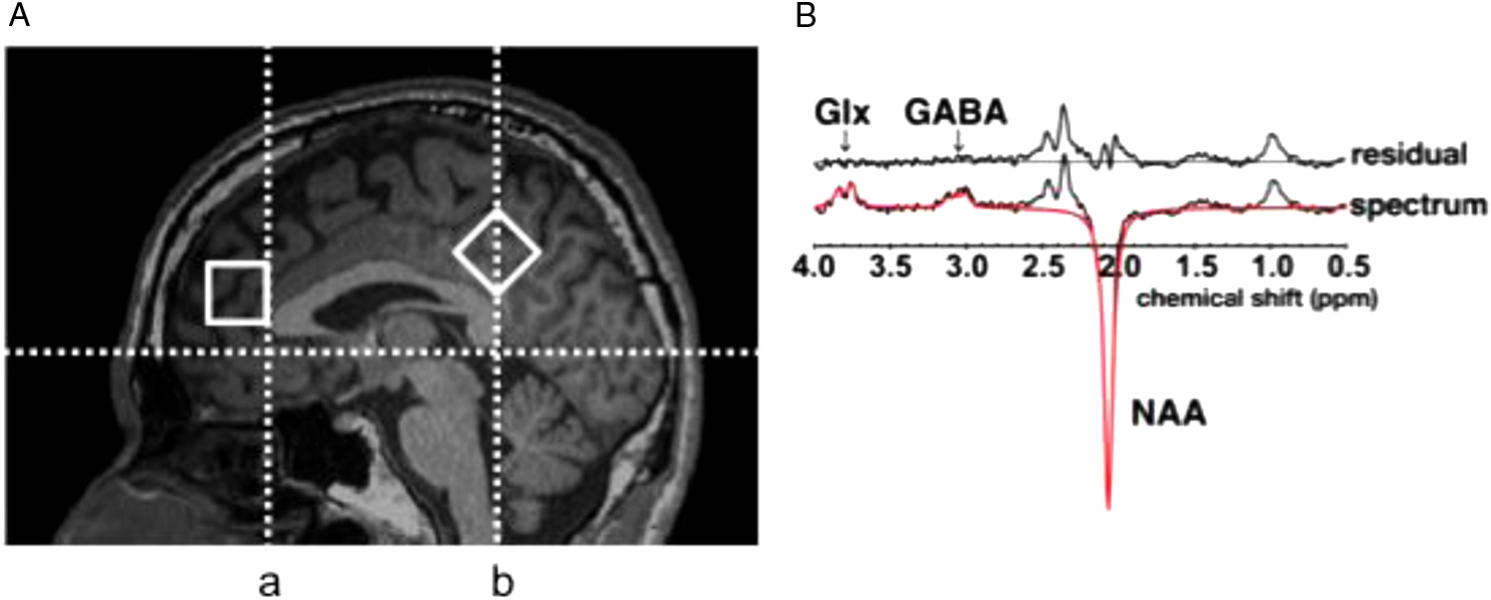
Magnetic resonance spectroscopy (MRS) using MEGA-PRESS. The volumes of interest (30 × 20 × 20 mm^3^) in MRS, which were located on mPFC and PCC, are shown in A; “line a” is set exactly on the rostral margin of the corpus callosum as the perpendicular axis to the anterior commissure–posterior commissure (AC–PC) line. “line b” is drawn as the perpendicular axis through the anterior commissure to the AC–PC line. The edited spectrum (i.e. black line) and fitted curve (i.e. red line), which were obtained for the signal quantification of GABA, Glx, and Cr levels, are shown in B.

Edited GABA and Glx MR spectra were acquired using the MEGA-PRESS sequence (Mescher *et al.*, 1998) with the following acquisition parameters: TR = 2400 ms; TE = 68 ms; number of averages = 512 for the mPFC and 256 for PCC. In both regions, the prescribed MRS VOIs was 30 × 20 × 20 mm^3^. Based on the chemical shift difference between the 3 ppm GABA resonance and the 3.75 ppm Glx resonance, the chemical shift displacement between GABA and Glx in the direction of the excitation pulse (bandwidth =3708 Hz, selective in the left–right direction), was 0.75 mm. In the directions of refocusing pulses (bandwidth = 1106 Hz, selective in the anterior–posterior direction and in the superior–inferior direction), the chemical shift displacement between GABA and Glx was 1.67 mm. The VOI in mPFC was defined as follows. After drawing “line a” exactly on the rostral margin of the corpus callosum as the perpendicular axis to the anterior commissure–posterior commissure (AC–PC) line (see Fig. 1A), the VOI in mPFC was set along “line a” and on the inferoposterior corner located at the rostral edge of the genu. The VOI in PCC was set above the superior surface of the corpus callosum together with the diagonal line of the VOI aligned along “line b,” which is the perpendicular axis across the posterior edge of the splenium to the AC–PC line (see Fig. 1A).

We measured the full-width at half-maximum (FWHM) of *N*-acetyl aspartate (NAA) peaks in the MEGA-PRESS spectra to determine the quality of shimming. The means ± standard deviations (SDs) were 6.864 ± 2.272 Hz in mPFC and 5.201± 2.203 Hz in PCC. We excluded the samples whose FWHM values of NAA peaks were large (i.e. FWHM > mean ± 2 SD). Although data from PCC were successfully collected from all the participants, those from mPFC of one participant were excluded from the analysis because the FWHM of the NAA peak was larger than the cut-off value. In addition, we evaluated motion artifacts by visual inspection of superimposed spectra for all the excitations during scanning using MEGA-PRESS. As a result, two samples obtained from mPFC were also excluded owing to motion artifacts.

#### DTI and T1-weighted anatomical imaging data acquisitions

DTI data of the participants were acquired using a clinical 3.0 Tesla Prisma MRI scanner (Siemens, Erlangen, Germany) with a 12-channel head coil. The entire brain was scanned by echo-planar imaging with 30 noncolinear motion probing gradients at a b-value of 1000 sec/mm^2^ and 5 T_2_-weighted b = 0 images. The acquisition parameters for the DTI scan were as follows: TE = 84 ms, TR = 6500 ms, 50 axial slices, slice thickness = 3 mm, field of view = 23 × 23 cm^2^, and matrix size = 128 × 128 (i.e. voxel size = 1.8 × 1.8 × 3.0 mm^3^). To improve signal-to-noise ratio, the scanning was repeated twice [number of excitation (NEX) = 2].

We also obtained high-resolution T1-weighted images for GM morphometry. MP-RAGE sequencing yielding 176 continuous slices of 1.0 mm thickness was carried out along the sagittal plane. The acquisition parameters were as follows: echo time = 2 ms; repetition time = 2000 ms; inversion time = 990 ms; flip angle = 9°; field of view = 256 mm; matrix size = 256 × 256; voxel size = 1 × 1 × 1 mm^3^.

### Processing and analysis of MRS and DTI data

#### Measurement of Gaba and GLX Levels by MRS

Prior to signal averaging, each scan was frequency- and phase-aligned by spectral registration (Near *et al.*, 2015) using the FID-A toolkit (https://github.com/cic-methods/fid-a) in MATLAB (MathWorks, Natick, MA, USA) to minimize the effects of frequency and phase drifts. Motion-corrupted averages were removed as described previously (Near *et al.*, 2013; Simpson *et al.*, 2017). Averaged difference spectra and sum spectra were then line-broadened using a 5-Hz Lorentzian filter, and zero-order phase corrections were applied manually to ensure upright peaks. First-order phase corrections were also applied manually in some cases because the first point in the FID did not always correspond exactly to the top of the echo. Following preprocessing, GABA and Glx signals from the difference spectra and creatine (Cr) signals from the sum spectra were quantified using the AMARES package provided in jMRUI software (Naressi *et al.*, 2001; Vanhamme *et al.*, 2001). GABA, Glx, and Cr were modeled as a triplet, a doublet, and a singlet of Lorentzian peaks, respectively. Because the editing efficacy for Glx has not been determined in the current sequence, Glx signals are shown in an arbitrary unit value.

#### Measurement of MD Within MRS VOIs

Diffusion tensor data were converted into the NIfTI format using MRI Convert (http://lcni.uoregon.edu/~jolinda/MRIConvert). The data were preprocessed using FMRIB Software Library (FSL) version 4.1.5 (http://www.fmrib.ox.ac.uk/fsl). This procedure included the following: (1) eddy current correction, (2) motion correction by registering all the diffusion-weighted data to the b = 0 images, which were corrected first, (3) brain extraction, (4) calculation of diffusion tensor and diagonalization, and (5) transformation to the MNI space. After these processes, the MD map in the MNI space was automatically constructed using FSL.

**Figure 2.**
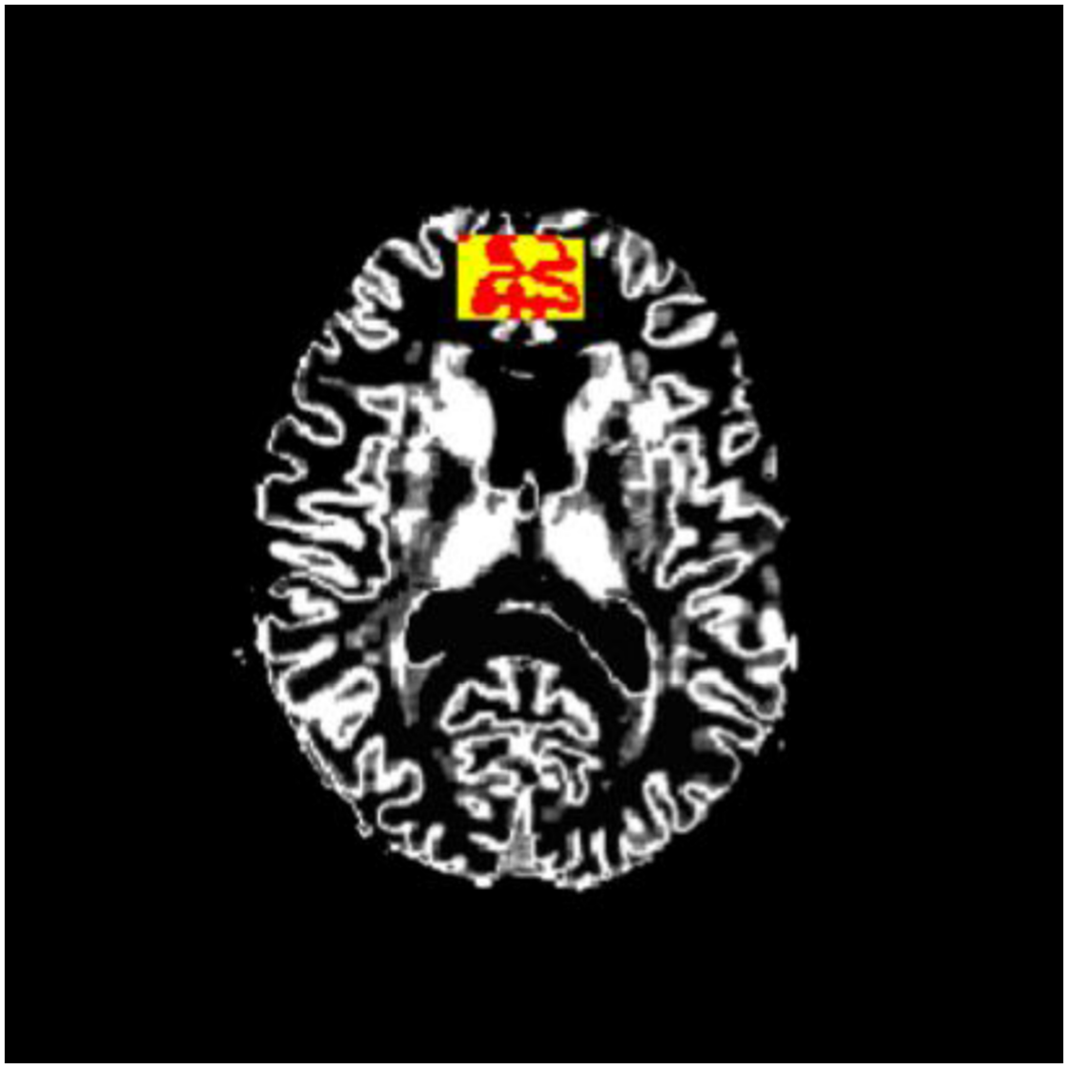
Binary mask of GM within mPFC VOI for measurement of MD. The figure shows a segmented GM image together with mPFC VOI in MRS (yellow) and a binary mask of GM within mPFC VOI in MRS (red) in the original space.

Then, the obtained T1-weighted anatomical images were segmented into GM, WM, and cerebrospinal fluid (CSF) using FAST (FMRIB’s automated segmentation tool) (Zhang *et al.*, 2001) in FSL software (available from http://fsl.fmrib.ox.ac.uk.proxy.bib.uottawa.ca/fsl/fslwiki/) (Jenkinson *et al.*, 2012; Smith *et al.*, 2004; Woolrich *et al.*, 2009; Zhang *et al.*, 2001) to calculate the relative volume of each tissue within MRS VOIs. After the binary GM masks within MRS VOIs were formed from segmented GM images and MRS VOIs (Fig. 2), Statistical Parametric Mapping (SPM; http://www.fil.ion.ucl.ac.uk/spm/) was used to coregister different spaces between the binary GM masks and the MD map in each subject. The threshold of GM mask was set to the default SPM parameter of 0.8. Because MD is markedly higher in CSF than in the brain tissue, the map of MDs lower than, 0.102 × 10^−2^ mm^2^/s was generated to attenuate the CSF effect on MD measurement within MRS VOIs, in accordance with the previous method described by Albrecht *et al.* (2007), i.e. the above-mentioned cut-off values were set at 3 SDs above the mean measured tissue MD. Finally, we calculated mean MD from nonzero voxels of the MD map within binary GM masks of MRS VOIs, i.e. mPFC and PCC.

### Statistical analysis

The metabolite ratios are presented in the results section of this study, i.e. raw GABA/Cr and Glx/Cr, and GM corrected GABA/Cr and Glx/Cr, which were divided by the relative volume of GM within each VOI in MRS in accordance with previous studies (Fujihara *et al.*, 2015; Jocham *et al.*, 2012). To assess the relationship among demographic characteristics, CANTAB scores, and metabolite ratios in MRS, Pearson’s correlation test were performed. In addition, we also performed regression analysis, in accordance with a previous report (Yildiz *et al.*, 2014). A p-value of < 0.05 was set as statistically significant. Student’s t-test was conducted to estimate gender differences in demographic characteristics. All the statistical analyses were carried out using SPSS version-24 (IBM Corporation).

## Results

All the subjects showed MoCA scores ≥ 20, and the predicted IQs were higher than 90 in all the participants (mean ± SD = 112.2 ± 7.6; see Table 1). Pearson’s correlation test showed that in mPFC and PCC, NAA level, Cr level, raw GABA/Cr or Glx, or GM corrected GABA/Cr or Glx/Cr did not significantly correlate with age, education years, predicted IQ, MoCA score, or CANTAB test scores, i.e. SRM and RVIP scores. Furthermore, MD in mPFC and PCC did not significantly correlate with age, education years, predicted IQ, MoCA score, or the above-mentioned CANTAB test scores. The MD in mPFC or PCC did not correlate with Cr level in each VOI, respectively.

**Figure 3.**
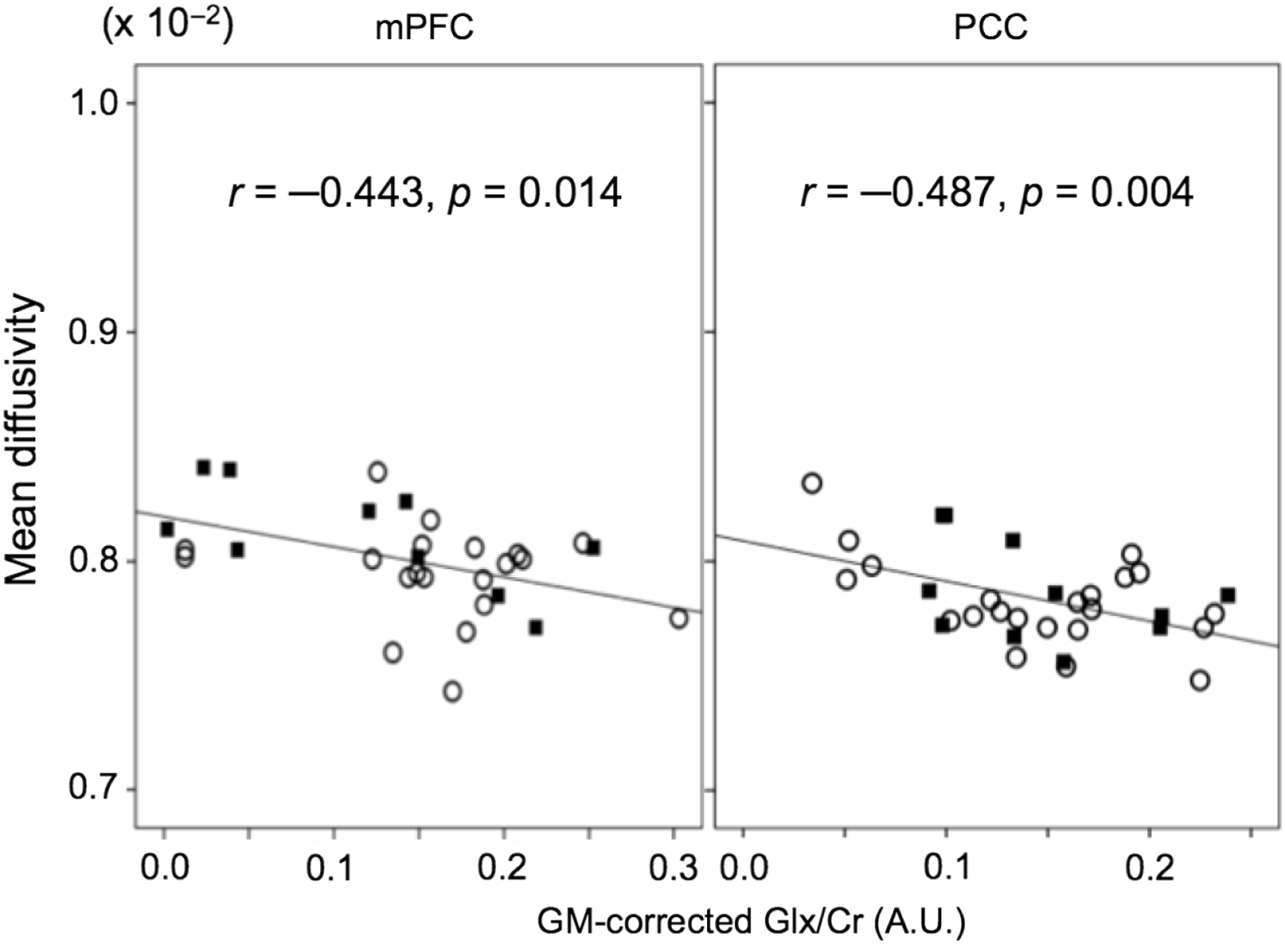
Scatter plots showing correlation between cortical MD and GM corrected Glx/Cr in mPFC and PCC. Squares indicate male subjects and circles indicate female subjects.

The raw Glx/Cr levels in mPFC and PCC were significantly negatively correlated with MD in each VOI (*r* = −0.477, *p* = 0.008 and *r* = −0.486, *p* = 0.004, respectively). The GM corrected Glx/Cr levels in mPFC and PCC were also significantly negatively correlated with MD in each VOI (*r* = −0.443, *p* = 0.014 and *r* = −0.487, *p* = 0.004, respectively) (Fig. 3). No significant correlations were observed between MD in mPFC and Glx level in PCC and between MD in PCC and Glx level in mPFC. Furthermore, raw or GM corrected GABA/Cr in mPFC and PCC did not significantly correlate with MD in either VOI.

In regression analysis, the raw Glx/Cr in mPFC and PCC could predict MD in each VOI (*β* = −0.477, t = −2.871, *p* = 0.008, and *β* = −0.486, t = −3.94, *p* = 0.004). Also, the GM corrected Glx/Cr in mPFC and PCC could predict MD in each VOI (*β* = −0.443, t = −2.617, *p* = 0.014, and *β* = −0.487, t = −3.102, *p* = 0.004). To control further for possible effects of GM, WM and CSF fractions were used in separate regression models for raw Glx/Cr, as reported by Fujihara *et al.* (2015) and Yildiz *et al.* (2014). All these models revealed that neither GM, WM, nor CSF explained the further variance in the models (mPFC: |*β*| < 0.463, |*t*| < 1.064, *p* > 0.297; and PCC: |*β*| < 0.131, |*t*| < 0.617, *p* > 0.542). These regression models showed that only raw Glx/Cr has a predictive effect on MD in each VOI (mPFC: *β* = −0.471, t = −2.724, *p* = 0.011; and PCC: *β* = −0.478, t = −2.908, *p* = 0.007). Also, GM corrected Glx/Cr has a predictive effect on MD in each VOI (mPFC: *β* = −0.422, t = −2.68, *p* = 0.021; and PCC: *β* = −0.477, t = −2.975, *p* = 0.006).

## Discussion

The results of this study show that cortical MD is significantly negatively correlated with Glx levels in both mPFC and PCC in healthy elderly individuals. On the other hand, no significant association was found between GABA level and cortical MD in both VOIs.

Glx level is very likely related to excitatory neurotransmission, because glutamate is considered to be the major component of the Glx signal (Bauer *et al.*, 2013; Fujihara *et al.*, 2015). The dominant pathway for glutamine production is via metabolism of neurotransmitter glutamate, so Glx represents the integrated metabolic and neurotransmitter functions of glutamate in the brain (Jahng *et al.*, 2016; Yuksel and Ongur, 2010). The glutamate level in a certain GM region is suggested to be associated with neuronal dysfunction and loss in that GM region (Segovia *et al.*, 2001). Thus, previous MRS studies showed that a decreased Glx level can be observed in neurodegenerative conditions, such as aging (Goryawala *et al.*, 2016; Grachev *et al.*, 2001), mild cognitive impairment and Alzheimer’s disease (Antuono *et al.*, 2001; Huang *et al.*, 2016; Riese *et al.*, 2015), in several GM regions including mPFC and PCC, which implies neuronal dysfunction and loss in these GM regions. Consistently, cognitive performance or cognitive symptoms have been reported to positively correlate with Glx levels in elderly people (Zahr *et al.*, 2008) and patients with mild cognitive impairment (Nikolova *et al.*, 2017) and Alzheimer’s disease (Walecki *et al.*, 2011). Then, an increased cortical MD, which has been observed in aging and the above-mentioned diseases (Lin *et al.*, 2016; Ni *et al.*, 2010; Ray *et al.*, 2006; Weston *et al.*, 2015), may reflect the decline in the magnitude of microstructural organization in the GM associated with aging and age-related diseases, i.e. a breakdown of cytoarchitectural barriers such as the cell membrane or a shift in the concentration of water between intra- and extracellular spaces (Elman *et al.*, 2017; Neil *et al.*, 2002; Sundgren *et al.*, 2004; Van Camp *et al.*, 2012). Considering the above-mentioned previous reports on Glx level and cortical MD, the significant negative association between cortical MD and Glx level in mPFC and PCC observed in this study suggests that age-related neuronal loss, which can be detected on the basis of cortical MD in DTI, leads to the decline of Glx metabolism within the above-mentioned GM regions.

In this study, no significant association was found between GABA levels and cortical MD in both mPFC and PCC. Similarly to Glx levels, previous MRS studies have shown that the GABA levels in mPFC and PCC are lower in healthy elderly people and patients with Alzheimer’s disease and mild cognitive impairments than in controls (Bai *et al.*, 2015; Riese *et al.*, 2015). Moreover, cognitive dysfunction has been reported to be associated with decreased GABA levels in mild cognitive impairment and Alzheimer’s disease (Porges *et al.*, 2017). Although such observations suggest that not only Glx levels, but also GABA levels reflect age-related neuronal loss, this speculation is inconsistent with the findings of our study. There are some difficulties in explaining the different findings on Glx and GABA levels in this study, but an asymmetric degeneration of glutamatergic neurons and GABAergic neurons during aging might contribute to our results regarding GABA in this study, that is, terminals and synapses of glutamatergic neurons, not those of GABAergic neurons, are reported to be predominantly affected in early-stage Alzheimer’s disease (Huang *et al.*, 2016; Kashani *et al.*, 2008; Proctor *et al.*, 2010). Additional studies including age-related neurodegenerative disorders, i.e. mild cognitive impairment and Alzheimer’s disease, should be carried out. Furthermore, the lack of significant GABA observations might have been due to the small sample size in this study.

A major limitation in this study is its small sample size, which may have influenced our statistical results. In addition, owing to the lack of data from subjects with age-related neurodegenerative disorders, i.e. mild cognitive impairment or Alzheimer’s disease, there are some limitations in this study. Although some previous MRS studies have shown the significant associations between cognitive function test scores and the levels of several cortical metabolites, i.e. NAA, GABA or Glx (Jessen *et al.*, 2013; Porges *et al.*, 2017), we failed to find such significant associations, which may be due to the small number of subjects in this study. In addition, young subjects should also be enrolled. Further MRS studies with larger sample sizes including subjects of various ages and with age-related neurodegenerative disorders will reinforce our findings in the future.

In conclusion, the level of Glx, which reflects excitatory neurotransmission, was associated with cortical MD in mPFC and PCC in GM. These findings might support a hypothesis, i.e. neuronal loss detected on the basis of cortical MD in DTI can cause a decline of Glx metabolism in these GM regions in elderly people. The results of this study can lead to a better understanding of the association between microstructural organization and neuronal metabolism in GM during aging.
